# Plasma Profile of Brain‐Derived Tau After Ischemic Stroke

**DOI:** 10.1111/ene.70735

**Published:** 2026-08-19

**Authors:** Karl Sjölin, Björn Röyter, Bianka Forgo, Julia Aulin, Kim Kultima, Johan Lindbäck, Jakob O. Ström, Joachim Burman

**Affiliations:** ^1^ Department of Medical Sciences, Translational Neurology Uppsala University Uppsala Sweden; ^2^ Department of Obstetrics and Gynecology, Faculty of Medicine and Health Örebro University Örebro Sweden; ^3^ Department of Clinical Neuroscience Karolinska Institute Stockholm Sweden; ^4^ Department of Neuroradiology Karolinska University Hospital Stockholm Sweden; ^5^ Faculty of Medicine and Health Örebro University Örebro Sweden; ^6^ Department of Medical Sciences, Cardiology Uppsala University Uppsala Sweden; ^7^ Uppsala Clinical Research Center (UCR) Uppsala University Uppsala Sweden; ^8^ Department of Medical Sciences, Clinical Chemistry Uppsala University Uppsala Sweden; ^9^ Department of Neurology, Faculty of Medicine and Health Örebro University Örebro Sweden

**Keywords:** BD‐tau, biomarkers, stroke

## Abstract

**Background and Aim:**

Brain‐derived tau (BD‐tau) is a predominantly CNS‐derived form of tau protein detectable in blood that reflects brain injury and has emerged as a potential biomarker in ischemic stroke for assessing neuronal damage, monitoring infarct progression, and predicting functional outcome. However, its temporal profile and the optimal timing for correlation with infarct volume remain unclear.

**Methods:**

This prospective cohort study included patients with ischemic stroke, with venous blood samples collected daily during the first week and at 3 months after symptom onset. Infarct volume was assessed by MRI at 48–72 h. Plasma BD‐tau concentrations were measured using a Simoa assay. Linear mixed‐effects models were used to analyze changes in biomarker levels. The optimal time point for correlation between BD‐tau and infarct volume was identified using a 24‐h moving‐window Pearson correlation analysis.

**Results:**

Fifty‐four patients (median age 78 years, 50% female) contributed 285 plasma samples. Plasma levels of BD‐tau followed a parabolic trajectory reaching a peak approximately 5 days after symptom onset. After 90 days, the concentration of BD‐tau was lower than in the last measurement of the first week. The strongest correlation between BD‐tau and infarct volume occurred at 105 h (4.4 days) (*r* = 0.89, 95% CI: 0.78–0.95) after symptom onset.

**Conclusions:**

Plasma BD‐tau exhibits a distinct temporal profile after ischemic stroke, with peak levels and maximal correlation with infarct volume occurring around Day 4–5. These findings have important implications for interpreting BD‐tau levels and optimizing sampling strategies in clinical studies.

**Trial Registration:**

Clinicaltrials.gov NCT03812666

## 1 Introduction

Blood‐based biomarkers have the potential to add biological precision to clinical decision‐making across the stroke care continuum [1]. Biomarkers of neuroglial injury have shown accuracy in stroke subtype diagnosis, outcome prediction, and prognosis, and we and others have reported the value of these types of biomarkers, such as glial fibrillary acidic protein, neurofilament light, and tau [2, 3, 4]. Previous analyses of tau in this setting have focused on total tau, which lacks specificity due to the influence of peripheral tau isoforms [5]. Brain‐derived tau (BD‐tau) is a predominantly CNS‐derived form of tau protein detectable in blood that has emerged as a biomarker reflecting brain injury in stroke, neurodegenerative, and traumatic brain injury [6, 7, 8]. Recent studies indicate that BD‐tau is elevated in stroke patients compared to mimics, correlates strongly with final infarct volume, and can track ischemic brain injury over time, outperforming both neuroimaging and clinical variables in predicting functional outcome [6, 9, 10, 11, 12].

However, the number of studies of BD‐tau in ischemic stroke remains limited, and its temporal dynamics are unclear. In this study, we aimed to characterize the plasma profile of BD‐tau during the first week after ischemic stroke, and to determine the time point at which BD‐tau shows the strongest association with infarct volume.

## 2 Materials and Methods

### 2.1 Ethical Approval and Informed Consent

This study adhered to the principles of the Declaration of Helsinki and received approval from the Regional Ethical Review Board in Uppsala, Sweden (Dnr: 2018/288), as well as from the Swedish Ethical Review Authority (Dnr: 2020–06000). All patients provided written informed consent, except those unable to do so due to aphasia or other cognitive impairments. In those cases, consent was obtained from their next of kin.

### 2.2 Study Design, Participants, and Data Collection

This is a substudy of the Inflammatory Biomarkers In Stroke (IBIS) observational study (NCT03812666). Detailed descriptions of study design, participant recruitment, sample handling, neuroimaging procedures, and statistical modeling have been published previously [4, 13]. In brief, the IBIS study prospectively enrolled patients with acute stroke admitted to the stroke unit at Örebro University Hospital, Sweden, from 2019 to 2022. Patients meeting the study criteria were consecutively invited to participate, although enrollment was temporarily paused during the COVID‐19 pandemic. Eligibility required clinical symptoms and radiologic findings consistent with acute stroke, with symptom onset within 48 h before the first study blood sample collection. Exclusion criteria were complete symptom resolution within 24 h (i.e., transient ischemic attack, TIA), contraindications to magnetic resonance imaging (MRI), or active inflammatory disease. Venous blood samples were collected daily during the hospital stay and again at a 3 month follow‐up visit. Samples were aliquoted and stored at −80°C for later analysis. While the original study included both hemorrhagic and ischemic strokes, this analysis focused solely on the subset of patients with ischemic stroke. MRI was performed 48–72 h after admission, including axial T2 and DWI sequences. Infarct volume was quantified using MRIcron with semi‐automatic segmentation, followed by manual correction when needed, as previously described [14].

### 2.3 Biochemical Analysis

Plasma BD‐tau concentrations were measured with Single Molecule Array (Simoa) using the BD‐Tau Advantage PLUS Assay on the SR‐X Biomarker Detection System (Quanterix Corp., MA, USA). The lower limit of quantification was 0.133 pg/mL, and the lower limit of detection was 0.044 pg/mL. All analyses were performed in batches by the same certified technician at the Department of Clinical Chemistry, Uppsala University Hospital, Sweden.

### 2.4 Statistical Analysis

The overall modeling framework of biomarker kinetics has been reported previously [4]. All time‐dependent analyses were performed relative to time from symptom onset. The temporal variation in BD‐tau levels was examined using a linear mixed‐effects model, with a restricted cubic spline for time included as a fixed effect and individual intercepts and slopes of the curve as random effects. The influence of infarct volume on BD‐tau levels was quantified as the reduction in interindividual variance of BD‐tau concentrations when adding infarct volume to the model. To identify the timepoint with the strongest correlation between BD‐tau levels and infarct volume, Pearson correlation coefficients were calculated for all observations within a ±12‐h window around each plotted time point, generating a 24‐h moving‐window correlation curve. An ordinal regression model was fitted to assess the association between BD‐tau and the proportion of gray matter in the infarcted area. The model included the proportion of gray matter as outcome and log (BD‐tau) as a linear term. Statistical significance refers to two‐sided tests at the 5% level. R version 4.4.3 RC and GraphPad Prism v 10.4.2 were used for all analyses.

## 3 Results

### 3.1 Clinical Characteristics

In total, 54 patients with a final diagnosis of ischemic stroke were included. Median age was 78 (interquartile range, IQR 72–83), 50% were female, and the median infarct volume was 5.5 (1.4–20) cm^3^. NIHSS at inclusion was 4 (2–10), and the most common comorbidities were hypertension (67%), atrial fibrillation (39%), hyperlipidemia (26%), and prior stroke (26%). MRI data were missing in five patients. Eight patients (15%) received intravenous thrombolysis, and 5 patients (9%) received endovascular thrombectomy.

### 3.2 Temporal Profile and Correlation With Infarct Volume

**FIGURE 1:**
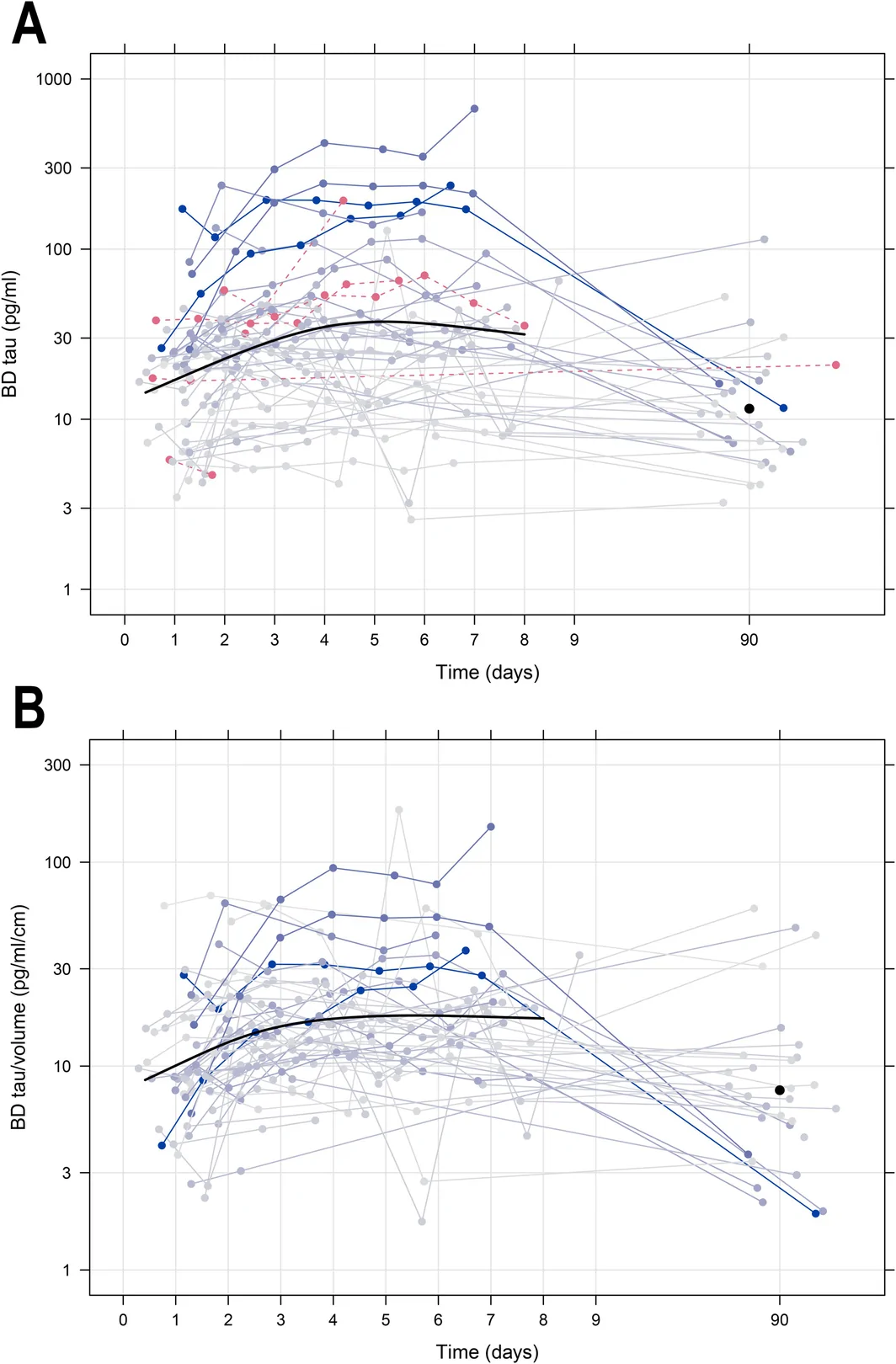
*Longitudinal changes in plasma BD‐tau concentrations during the first week following ischemic stroke*. Individual trajectories of plasma BD‐tau are displayed as blue lines, with increasing color intensity reflecting larger infarct volumes. The fitted black curve represents the estimated population trajectory obtained from a restricted cubic spline model, whereas the black marker denotes the mean concentration at the 90‐day follow‐up. (A) Analysis including all participants (*n* = 54); individuals lacking MRI‐derived infarct volumes (*n* = 5) are shown with red dashed lines. (B) Estimated trajectory after accounting for infarct volume (*n* = 49).

**FIGURE 2:**
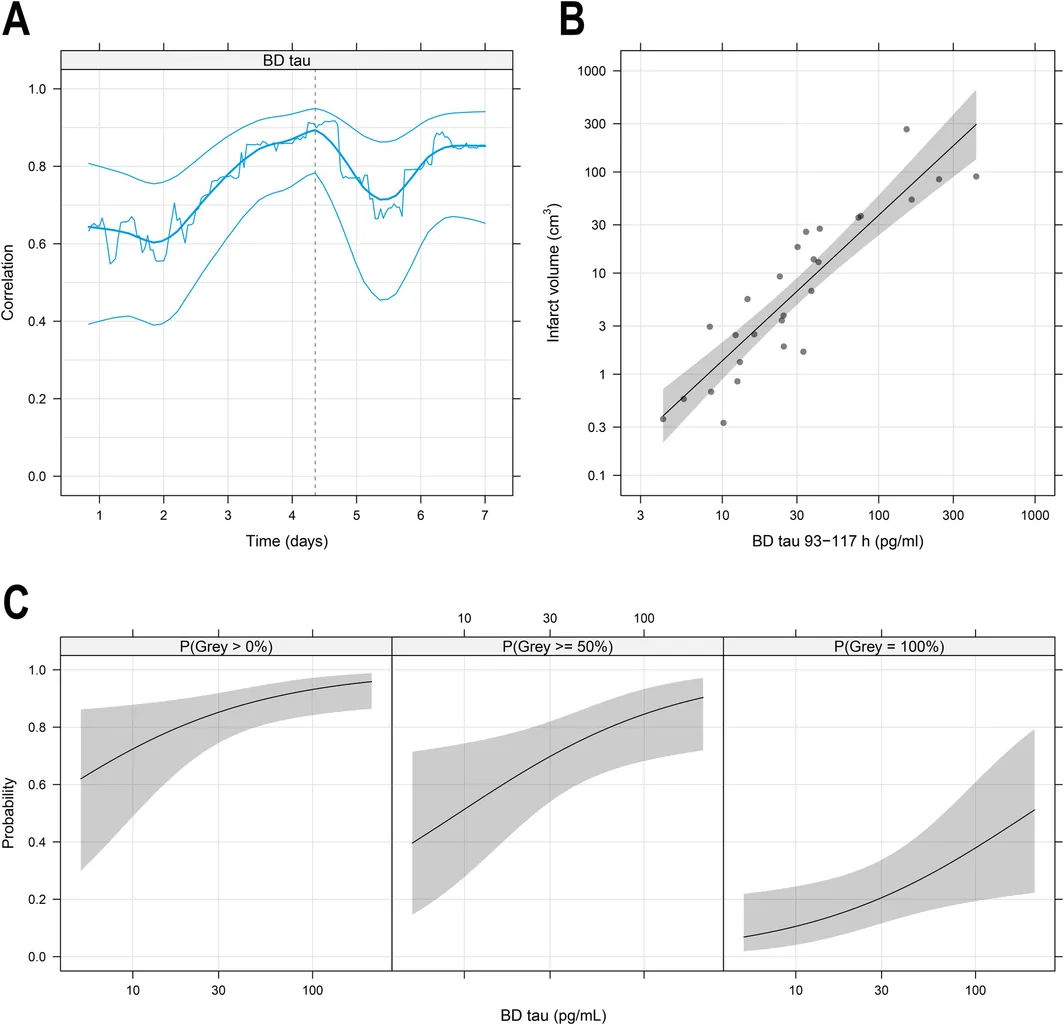
*Relationship between plasma BD‐tau, infarct volume, and gray matter involvement*. (A) Time‐dependent association between infarct volume and plasma BD‐tau concentration during the first week after stroke. The blue curve depicts moving‐window Pearson correlation coefficients, with shaded limits indicating the corresponding 95% confidence intervals. Peak correlation was observed 105 h after symptom onset (vertical dashed line). (B) Scatterplot illustrating the relationship between infarct volume and plasma BD‐tau concentration at the time point of maximal correlation. The solid line represents the fitted regression model and the shaded area its 95% confidence interval. (C) Estimated probability of gray matter involvement according to plasma BD‐tau concentration. The panels show the probability that the infarct includes any gray matter (left), ≥ 50% gray matter (middle), or exclusively gray matter (right), based on the first BD‐tau measurement obtained between Days 3 and 6 (*n* = 37). For instance, BD‐tau concentrations above 100 pg/mL corresponded to an estimated probability of ~85% that at least half of the infarct involved gray matter.

Patients provided blood samples at a median of 5.5 (3–7) time points, with the first collected at 30 (23–39) hours after symptom onset. Altogether, 285 blood samples were analyzed. Plasma levels of BD‐tau followed a parabolic trajectory peaking at 5.2 days (Figure 1A). Patients with infarcts in the brain stem or cerebellum tended to have lower levels of BD‐tau than patients with infarcts located supratentorially. Adjustment for infarct volume attenuated the parabolic shape, but the peak timing was essentially unchanged (Figure 1B). After adjusting for volume, the interindividual variance in BD‐tau levels was reduced by 59% (patients without MRI data [*n* = 5] were not included in this analysis). The strongest correlation between BD‐tau and infarct volume occurred at 105 h (4.4 days) (*r* = 0.89, 95% CI: 0.78–0.95) after symptom onset (Figure 2A,B). After 90 days, the concentration of BD‐tau was lower than in the last measurement of the first week (Figure 1).

### 3.3 Gray vs. White Matter

For a doubling of BD‐tau, the relative odds of having a larger proportion of gray matter within the infarct were 1.64, 95% CI: 1.08–2.50, *p* = 0.02. The probability that ≥ 0%, ≥ 50%, or = 100% of the infarct involved gray matter at any given BD‐tau level was illustrated graphically (Figure 2C).

## 4 Discussion

In this study, BD‐tau exhibited a parabolic trajectory, peaking on day 5 and showing the strongest correlation with final infarct volume 4 days after symptom onset. These findings provide a more precise characterization of BD‐tau temporal dynamics than previously reported and may help inform optimal timing of biomarker sampling in both clinical and research settings.

The temporal profile of BD‐tau has been partially described in two previous studies, showing increasing levels up to 72 h and Day 7, particularly in patients with poor outcomes or large infarcts [10, 12]. However, neither study assessed Days 4–6, potentially explaining why peak BD‐tau levels were not identified. Two studies have reported strong associations between BD‐tau and infarct volume, with correlations strengthening over time [6, 12]. Peak associations were observed around Days 2–3 in those studies, while our findings indicate a maximum after Day 4. Together, these results suggest that the relationship between BD‐tau and infarct volume increases and may peak mid‐week after stroke onset.

When BD‐tau levels were adjusted for infarct volume, interindividual variance decreased by 59%. Thus, a substantial proportion of the variation in BD‐tau can be explained by differences in infarct volume. Nevertheless, a considerable portion (41%) is attributable to other factors. Given that tau is predominantly expressed in cortical neurons, lesion location likely contributes to residual variability in BD‐tau levels [15]. This is supported by our exploratory analysis linking BD‐tau to gray matter involvement and by a previous study showing that BD‐tau levels correlated with infarct diameter in gray but not white matter [11]. Differential susceptibility to ischemia in gray and white matter is also likely to contribute to this pattern, especially in patients receiving successful reperfusion therapy.

Several limitations should be noted. First, only 54 patients were included, limiting extensive adjustment for clinical parameters. Second, few samples were collected in the hyperacute time window, restricting any conclusions regarding this period. Third, BD tau may have a biphasic kinetic profile extending beyond the first week, and thus not captured in this study. Fourth, temporal patterns may vary depending on infarct location, which was not accounted for. Fifth, the COVID‐19 pandemic led to substantial loss to follow‐up, and we therefore refrained from clinical outcome analyses. Sixth, no adjustment or direct comparison with other relevant neuroglial biomarkers was performed.

Despite these limitations, this high‐resolution serial sampling revealed a parabolic trajectory of BD‐tau during the first week after ischemic stroke, with the highest correlation to infarct volume occurring after 4 days, and an association with gray matter involvement in the infarct, further supporting the potential role of BD‐tau as a dynamic biomarker of brain injury in ischemic stroke.

## Author Contributions

**Karl Sjölin:** writing – original draft, conceptualization, methodology, formal analysis, project administration, funding acquisition, data curation. **Björn Röyter:** conceptualization, investigation, writing – review and editing, data curation, project administration. **Bianka Forgo:** conceptualization, investigation, writing – review and editing. **Julia Aulin:** writing – review and editing. **Kim Kultima:** writing – review and editing, resources. **Johan Lindbäck:** methodology, visualization, formal analysis, writing – review and editing. **Jakob O. Ström:** project administration, conceptualization, investigation, funding acquisition, writing – review and editing, supervision, resources. **Joachim Burman:** conceptualization, funding acquisition, writing – review and editing, methodology, resources, supervision.

## Funding

This work was supported by Regionala Forskningsrådet Uppsala/Örebro, Bissen Brainwalk Foundation, Nyckelfonden Research Committee at Örebro University Hospital and Swedish state support for research (ALF agreement).

## Conflicts of Interest

K.S. reports receiving speaker honoraria from Novo Nordisk and Amgen. J.A. reports receiving speaker honoraria from Alnylam. J.O.S. received fees for participating in scientific advisory boards for Bayer in 2016 and 2019 and has been hired as an adjudicator in a trial initiated by Astra Zeneca in 2024. B.R., B.F., J.L., K.K. and J.B. report no competing interests.

## Data Availability

Raw data are not publicly available to preserve individuals’ privacy under the European General Data Protection Regulation. A fully anonymized dataset supporting this article is available upon reasonable request to the corresponding author, provided that data transfer complies with EU legislation and decisions by the Swedish Ethical Review Authority.

## References

[1] S. Tiedt , A. Bustamante , A. Cameron , et al., “Biomarkers for Advancing Diagnosis and Prognosis in Stroke,” Lancet Neurology 25 (2026): 406–420, 10.1016/S1474-4422(25)00480-6. 41864237

[2] F. Pujol‐Calderon , H. Zetterberg , E. Portelius , et al., “Prediction of Outcome After Endovascular Embolectomy in Anterior Circulation Stroke Using Biomarkers,” Translational Stroke Research 13 (2022): 65–76, 10.1007/s12975-021-00905-5. 33723754 PMC8766380

[3] M. Correia , I. Silva , D. Gabriel , et al., “Early Plasma Biomarker Dynamic Profiles Are Associated With Acute Ischemic Stroke Outcomes,” European Journal of Neurology 29 (2022): 1630–1642, 10.1111/ene.15273. 35124870

[4] K. Sjölin , B. Royter , B. Forgo , et al., “Plasma Profiles of Neuroglial Injury Biomarkers After Ischemic Stroke,” Translational Stroke Research 16 (2025): 2185–2194, 10.1007/s12975-025-01380-y. 40900222 PMC12598674

[5] F. Gonzalez‐Ortiz , M. Turton , P. R. Kac , et al., “Brain‐Derived Tau: A Novel Blood‐Based Biomarker for Alzheimer's Disease‐Type Neurodegeneration,” Brain 146 (2023): 1152–1165, 10.1093/brain/awac407. 36572122 PMC9976981

[6] F. Gonzalez‐Ortiz , L. Holmegaard , B. Andersson , et al., “Plasma Brain‐Derived Tau Correlates With Cerebral Infarct Volume,” Journal of Internal Medicine 297 (2024): 173–185, 10.1111/joim.20041. 39639627 PMC11771704

[7] F. Gonzalez‐Ortiz , T. K. Karikari , G. M. Bentivenga , et al., “Levels of Plasma Brain‐Derived Tau and p‐tau181 in Alzheimer's Disease and Rapidly Progressive Dementias,” Alzheimer's & Dementia 20 (2024): 745–751, 10.1002/alz.13516. PMC10841678 37858957

[8] F. Gonzalez‐Ortiz , M. Dulewicz , N. J. Ashton , et al., “Association of Serum Brain‐Derived Tau With Clinical Outcome and Longitudinal Change in Patients With Severe Traumatic Brain Injury,” JAMA Network Open 6 (2023): e2321554, 10.1001/jamanetworkopen.2023.21554. 37399012 PMC10318474

[9] T. M. Stanne , F. Gonzalez‐Ortiz , C. Brannmark , et al., “Association of Plasma Brain‐Derived Tau With Functional Outcome After Ischemic Stroke,” Neurology 102 (2024): e209129, 10.1212/WNL.0000000000209129. 38545929 PMC10962917

[10] R. Varela , F. Gonzalez‐Ortiz , A. Dias , et al., “Plasma Brain‐Derived Tau in Prognosis of Large Vessel Occlusion Ischemic Stroke,” Stroke 55 (2024): 2353–2358, 10.1161/STROKEAHA.123.046117. 39051090

[11] J. K. Gundersen , F. Gonzalez‐Ortiz , T. Karikari , et al., “Neuronal Plasma Biomarkers in Acute Ischemic Stroke,” Journal of Cerebral Blood Flow and Metabolism 45 (2025): 77–84, 10.1177/0271678X241293537. 39450480 PMC11563507

[12] N. Vlegels , N. L. Knuth , K. A. Steiner , et al., “Brain‐Derived Tau for Monitoring Brain Injury in Acute Ischemic Stroke,” Science Translational Medicine 18 (2026): eadz1280, 10.1126/scitranslmed.adz1280. 41533774

[13] B. Royter , N. Andreasson , B. Forgo , J. Kallman , and J. O. Strom , “Inflammatory Response After Stroke‐A Clinical Observation Study,” BMC Neurology 25 (2025): 233, 10.1186/s12883-025-04244-y. 40447994 PMC12125930

[14] C. Rorden , “MRIcron,” https://people.cas.sc.edu/rorden/mricron/index.html.

[15] K. Sjölin , K. Kultima , A. Larsson , et al., “Distribution of Five Clinically Important Neuroglial Proteins in the Human Brain,” Molecular Brain 15 (2022): 52, 10.1186/s13041-022-00935-6. 35765081 PMC9241296

